# Immediate replacement of fishing with dairying by the earliest farmers of the northeast Atlantic archipelagos

**DOI:** 10.1098/rspb.2013.2372

**Published:** 2014-04-07

**Authors:** Lucy J. E. Cramp, Jennifer Jones, Alison Sheridan, Jessica Smyth, Helen Whelton, Jacqui Mulville, Niall Sharples, Richard P. Evershed

**Affiliations:** 1 Organic Geochemistry Unit, School of Chemistry, University of Bristol, Cantock's Close, Bristol BS8 1TS, UK; 2 School of History, Archaeology and Religion, Cardiff University, Humanities Building, Colum Drive, Cardiff CF10 3EU, UK; 3 National Museums Scotland, Chambers Street, Edinburgh EH1 1JF, UK

**Keywords:** Neolithic diet, archaeology, pottery, biomarkers, lipids, stable carbon isotopes

## Abstract

The appearance of farming, from its inception in the Near East around 12 000 years ago, finally reached the northwestern extremes of Europe by the fourth millennium BC or shortly thereafter. Various models have been invoked to explain the Neolithization of northern Europe; however, resolving these different scenarios has proved problematic due to poor faunal preservation and the lack of specificity achievable for commonly applied proxies. Here, we present new multi-proxy evidence, which qualitatively and quantitatively maps subsistence change in the northeast Atlantic archipelagos from the Late Mesolithic into the Neolithic and beyond. A model involving significant retention of hunter–gatherer–fisher influences was tested against one of the dominant adoptions of farming using a novel suite of lipid biomarkers, including dihydroxy fatty acids, ω-(*o*-alkylphenyl)alkanoic acids and stable carbon isotope signatures of individual fatty acids preserved in cooking vessels. These new findings, together with archaeozoological and human skeletal collagen bulk stable carbon isotope proxies, unequivocally confirm rejection of marine resources by early farmers coinciding with the adoption of intensive dairy farming. This pattern of Neolithization contrasts markedly to that occurring contemporaneously in the Baltic, suggesting that geographically distinct ecological and cultural influences dictated the evolution of subsistence practices at this critical phase of European prehistory.

## 1. Introduction

The mechanism of the Neolithization of northwestern Europe has been debated for many years, with some arguing that Mesolithic inhabitants were prime movers, adopting domesticated animals and plants from their Continental neighbours, but retaining much of their own lifestyle [1]. Others argue that these domesticates were introduced as part of a novel package by immigrant farming groups from the Continent, followed by a rapid spread of Neolithic ideas [2,3]. In this ‘colonization’ scenario, a rapid acculturation of indigenous hunter–gatherer–fisher groups is envisaged, with key evidence derived from the stable carbon isotope signatures from Mesolithic and Neolithic human bone collagen. These reveal a marked difference in diet between Mesolithic and Neolithic coastal inhabitants, with enriched δ^13^C values in the former suggesting a significant marine protein component, while Neolithic individuals display predominantly terrestrial values [4,5].

While the faunal assemblages and strong marine isotopic signature in skeletal remains from coastal Late Mesolithic Britain are unambiguous, criticism has been levelled at the interpretation of low or non-existent contributions of marine products to the Neolithic diet. This is owing, in part, to the lack of sensitivity of the bulk collagen stable isotope approach for low-protein diets combined with possible scrambled routing of dietary carbon, which could render low quantities of marine protein (less than 20%) isotopically invisible [6,7]. Furthermore, it has been argued that possible Neolithic shell middens from Ireland and Scotland point to continued marine resource consumption, and the possibility has been raised that the skeletons investigated isotopically were not representative of the Neolithic population of Britain [6,8]; however, these critiques have been robustly rebutted [9].

In view of this controversy, we sought independent evidence based on the biomolecular and isotopic compositions of lipids preserved in prehistoric pottery from insular and coastal locations from the eastern North Atlantic, including mainland Britain, the Scottish isles and the isles of Man and Ireland (see electronic supplementary material, figure S1). This is a highly sensitive means of investigating the significance of marine product processing in pottery. Although the characteristic long-chain polyunsaturated fatty acid (PUFA) distributions of fresh marine fats and oils are lost from degraded marine lipids owing to rapid oxidation and polymerization, our recent work has identified more stable marine biomarkers, including ω-(*o*-alkylphenyl)alkanoic acids (APAAs) [10,11] and vicinal dihydroxy acids (DHYAs) [11,12] originating from the degradation of poly- and monounsaturated fatty acids, respectively. While APAAs require heating for formation [11,13], in the case of DHYAs heat is unnecessary because oxidation of monounsaturated fatty acids occurs spontaneously at room temperature [14]. These products preserve the carbon chain length (and, for the latter, double-bond positions) of precursor fatty acids and thus act as reliable proxies for the presence of products containing significant concentrations of long-chain PUFAs (i.e. marine or freshwater fats and oils). Although these products may exist only at low concentrations, operating the gas chromatograph/mass spectrometer (GC/MS) in selected ion-monitoring mode (SIM; see the electronic supplementary material, tables S1 and S2) greatly enhances the sensitivity of the analyses such that picogram per gram concentrations are routinely detectable.

Additionally, the determination of the δ^13^C values of *n*-alkanoic acids provides a robust signature of source(s) of dietary carbon and the metabolism of organisms. Specifically, ruminant species (cattle, sheep and goats) are separable from non-ruminants (e.g. pigs); milk fats, moreover, are separable from carcass fats, owing to more depleted δ^13^C values exhibited by octadecanoic acid in the former, resulting from its different biosynthetic origins [15]. Fats and oils of marine origin would exhibit higher δ^13^C values than terrestrial species, which is confirmed by our investigation of approximately 100 aquatic organisms from the North Atlantic (see the electronic supplementary material, figure S2) [13]. Together, these molecular and stable carbon isotope methods constitute a powerful multi-proxy approach for testing theories relating to marine and terrestrial resource processing in pottery vessels.

Our archaeological investigations focused on 1081 sherds and 142 associated carbonized deposits, yielding roughly 650 sufficiently well-preserved lipid residues for biomolecular and stable carbon isotopic analysis. A significant proportion of the sherds (more than 400; approx. 40%) derived from 48 Neolithic assemblages were chosen to increase sensitivity at this critical time. These sherds included pottery of: (i) Carinated Bowl tradition, before 3700 BC; (ii) the secondary expansion of the Neolithic to insular locations and Middle Neolithic Hebridean, ‘Unstan’ and early Grooved Wares, 3600–2900 BC; and (iii) later Neolithic Grooved and Ronaldsway Ware (2900–2300 BC; table 1; see the electronic supplementary material, figure S1). Post-firing heat discoloration, the incidence of sooting and the vessel shapes are diagnostic that these types of Neolithic vessels were commonly used for cooking, probably boiling. A longer chronological dimension was obtained from coastal and insular northern Britain, through the inclusion of 15 sites from the Bronze Age to Norse period. Existing evidence from faunal assemblages and human stable isotope information was also collated. This allowed three strands of proxy evidence for subsistence patterns to be aligned for this region, and hence chronological trends to be studied over 5000 years of prehistory.

**Table 1.** Pottery from coastal and island locations included in this study.

date	period	region	assemblages (*n*)	sherds (*n*)	visible deposits (*n*)	residues further analysed (*n*)
>4600 BC	Early Neolithic	Channel Isles	5	22	1	8
>3700 BC	Early Neolithic	mainland northern Britain, Isle of Man, island of Ireland	14	218	12	124
3600–2900 BC	Middle Neolithic	Outer Hebrides, Northern Isles, mainland northern Britain, island of Ireland	15	205	26	104
2900–2300 BC	Late Neolithic	Northern Isles, mainland northern Britain, Isle of Man, island of Ireland	14	272	28	172
2280–800 BC	Bronze Age	Outer Hebrides, Northern Isles, Isle of Man	6	79	8	40
800 BC–AD 800	Iron Age	Outer Hebrides, Northern Isles	6	162	25	100
AD 800–1400	Viking/Norse	Outer Hebrides, Northern Isles	3	123	42	95
*Σ*			63	1081	142	643

## 2. Material and methods

### (a) Solvent extraction

Any visible carbonized deposits were first removed using a solvent-cleaned scalpel. Small portions of the external surfaces of sherds were then cleaned using a modelling drill before the piece was removed using a chisel. Carbonized residues and cleaned sherd fragments were crushed in a solvent-washed mortar and pestle, and an internal standard added (10 or 20 μg *n*-tetratriacontane to the carbonized deposit or sherd fragment, respectively) prior to solvent extraction using 2 × 5 ml (carbonized residue) or 2 × 10 ml (sherd fabric) CHCl_3_/MeOH (2 : 1 v*/*v) via sonication (20 min). After centrifugation, the solvent was decanted and blown down to dryness under a gentle stream of N_2_.

### (b) Alkaline extraction

The ‘bound’ fraction from selected sherds was released through the alkaline extraction of solvent-extracted pottery using 5 ml 0.5 M NaOH/MeOH in DCM-extracted double-distilled water (9:1 v*/*v; 70°, 1 h). After acidification to pH 3 using 3 M aqueous HCl, ‘bound’ lipids were extracted using 3 × 3 ml DCM.

### (c) Preparation and analysis of trimethylsilyl ethers and esters

Aliquots of the solvent and alkaline extracts were filtered through a silica column and treated with 40 µl *N,O*-bis(trimethylsilyl)trifluoroacetamide (BSTFA) containing 1% trimethylsilyl chloride (70°, 1 h). These derivatives were analysed using high-temperature gas chromatography, using a GC fitted with a high-temperature non-polar column (DB1-HT; 100% dimethylpolysiloxane, 15 m × 0.32 mm i.d. 0.1 µm film thickness). The temperature programme comprised a 50°C isothermal hold followed by an increase to 350° at 10° min^−1^, followed by a 10 min isothermal hold. Alkaline extracts were immediately introduced via a split/splitless injector onto a GC/MS fitted with a non-polar column (100% dimethyl polysiloxane stationary phase; 60 m × 0.25 mm i.d. 0.1 µm film thickness). The instrument was a ThermoFinnigan single quadrupole TraceMS run in EI mode (electron energy 70 eV, scan time of 0.6 s). Samples were first run in full scan mode (*m/z* 50–650) and then SIM, scanning for the two cleavage fragments and [M-15]^+^ ions for the most common positional isomers of C_18_–C_22_ DHYAs (see electronic supplementary material, table S2) within the appropriate retention time windows. The temperature programme comprised an isothermal hold at 70° for 2 min, ramping to 220° at 10° min^−1^, followed by the second ramp at 4° min^−1^ to 300°, with a 10 min isothermal hold.

### (d) Preparation and analysis of fatty acid methyl esters

Aliquots of the total lipid extract were hydrolysed (0.5 M NaOH/MeOH; 70°, 1 h). The neutral fraction was removed (3 × 3 ml hexane) followed by acidification to pH 3 using 1 M aqueous HCl and the extraction of free fatty acids (3 × 3 ml CHCl_3_). Fatty acids were methylated using 100 µl BF_3_/MeOH (14% w/v, 75°, 1 h) and extracted (3 × 2 ml CHCl_3_). Fatty acid methyl esters were analysed using a GC/MS fitted with a polar column, with the MS operated in full scan (*m/z* 70–650) and SIM (*m/z* 105, 262, 290, 318 and 346 to determine APAAs) modes.

The δ^13^C values of individual fatty acids were determined using GC-combustion-isotope ratio MS (GC-C-IRMS). Analyses were performed using a Varian 3400 GC coupled to a Finnigan MAT Delta-S IRMS with a modified Finnegan MAT interface, Cu and Pt wires (0.1 mm o.d.) in an alumina reactor (0.5 mm i.d.). Samples were injected via an SPI injector onto a non-polar column (CP-Sil CB, 100% dimethylpolysiloxane, 50 m × 0.32 i.d. 0.1 µm film thickness). The temperature programme consisted of a 2 min isothermal hold at 50° and then ramped at 10° min^−1^ to 300°C followed by a 10 min isothermal hold. Results were calibrated against reference CO_2_, which was injected directly into the source three times at the beginning and end of the run. All samples were run in duplicate with external standards every four runs; any runs of unacceptable integrity were discarded and repeated. The δ^13^C values were derived according to the following expression and are relative to the international standard vPDB: δ^13^C‰ = ((*R*_sample_ – *R*_standard_)/*R*_standard_) × 1000, where *R* = ^13^C/^12^C. The δ^13^C values were corrected for the carbon atoms added during methylation using a mass balance equation [16].

## 3. Results and discussions

### (a) Marine biomarkers

**Figure 1.**
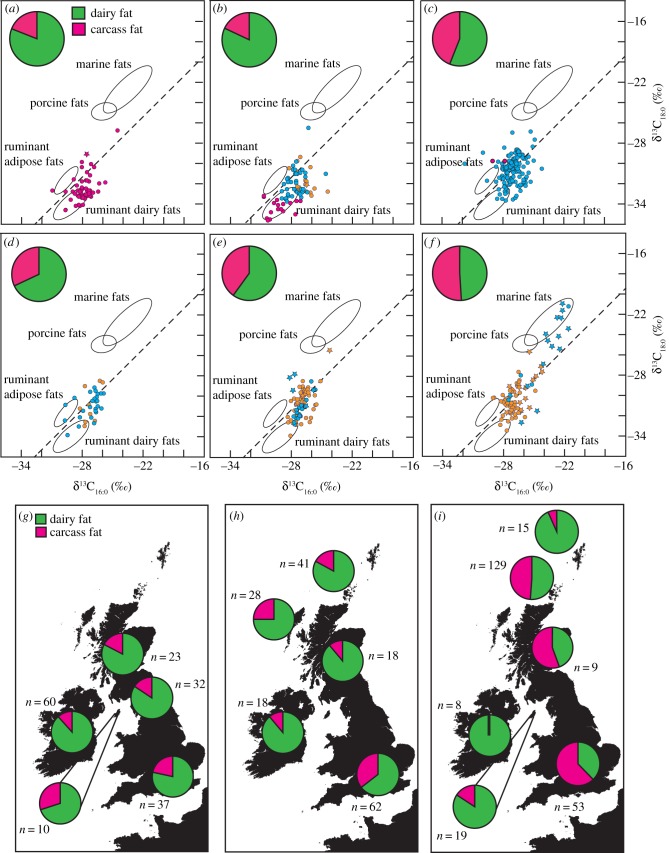
Prevalence of marine and dairy fats in prehistoric pottery determined from lipid residues. (*a*–*f*) Scatter plots show δ^13^C values determined from C_16:0_ and C_18:0_ fatty acids preserved in pottery from northern Britain (red circles), the Outer Hebrides (yellow circles) and the Northern Isles of Scotland (blue circles), dating to (*a*) Early Neolithic, (*b*) Mid/Secondary expansion Neolithic, (*c*) Late Neolithic, (*d*) Bronze Age, (*e*) Iron Age and (*f*) Viking/Norse. Star symbol indicates where aquatic biomarkers were also detected. Ellipses show 1 s.d. confidence ellipses from modern reference terrestrial species from the UK [19] and aquatic species from North Atlantic waters [13]. (*g*–*i*) Maps show the frequency of dairy fats in residues from Neolithic pottery from (*g*) Early Neolithic, (*h*) the Middle Neolithic/Secondary expansion and (*i*) Late Neolithic. Additional data from isotopic analysis of residues from Neolithic southern Britain (*n* = 152) and Scotland (*n* = 104) are included [19,20].

**Figure 2.**
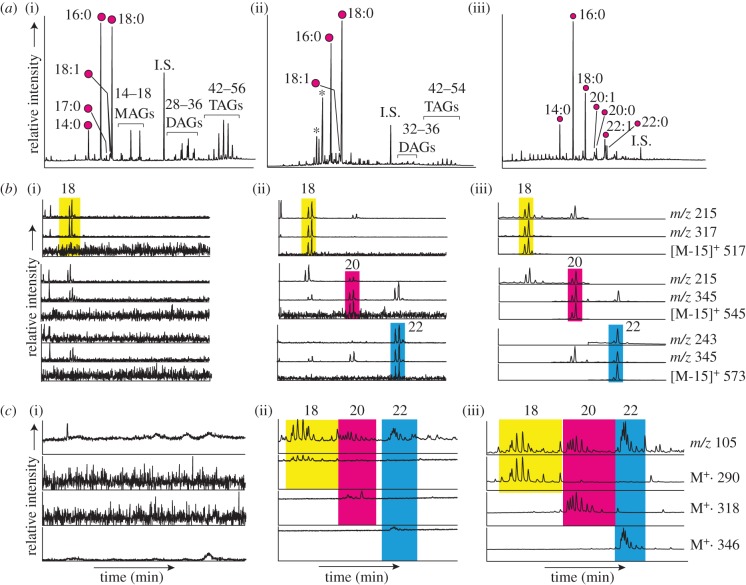
(*a*) Partial high-temperature gas chromatograms showing total lipid extracts from three sherds above mass chromatograms from aliquots analysed using GC/MS operated in SIM mode for the detection of (*b*) C_18_–C_22_ DHYAs (as tris-trimethylsilyl derivatives) and (*c*) C_18_–C_22_ APAAs (as methyl esters; electronic supplementary material, tables S1 and S2). Components were identified based upon the presence of identical chromatographic peaks for the characteristic fragment ions and molecular ions at the expected retention times. Residue (i) is from the Neolithic settlement at Braes o'Habreck on Wyre, the Orkney Isles; (ii) is from Cille Pheadair, South Uist; and (iii) is from Jarlshof, Shetland; only (ii) and (iii) contain detectable marine biomarkers, with (iii) displaying the strongest signatures. Filled circles represent free fatty acids with carbon number *x* and degree of unsaturation *y*. Retention window: (*a*) 5–35 min, (*b*) 23–30 min and (*c*) 24–36 min.

Over 300 Neolithic residues from coastal locations on northern Britain and the smaller isles of Orkney, Shetland, North and South Uist, Lewis and Man were investigated using high-sensitivity GC/MS-SIM. Lipid residues typically displayed compositions of saturated carboxylic acids consistent with degraded animal fats (see the electronic supplementary material, figure S3), with the incidence of a distinctive series of mid-chain ketones (e.g. approx. 30% of Early Neolithic residues) indicating that the fats extracted were regularly reaching temperatures in excess of 270°C [17,18]. Nonetheless, long-chain DHYAs and APAAs were detected in just two sherds, one from Moray on the Scottish mainland and one from South Uist on the Outer Hebrides (figure 1). The near-complete absence of aquatic biomarkers in the Neolithic pottery residues was supported by stable carbon isotope signatures of *n*-alkanoic acids, which reflect a predominantly ruminant origin for the majority of fats (figures 1*a–c* and 2; electronic supplementary material, table S3).

**Figure 3.**
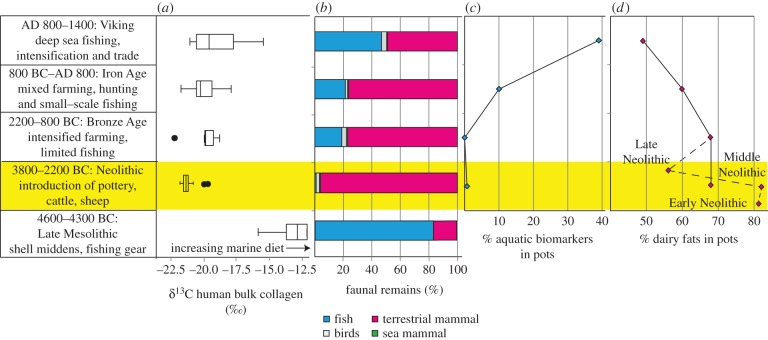
Multi-proxy palaeodietary information from the Western and Northern Isles of Scotland and mainland northern Britain. (*a*) Mean δ^13^C values from bulk human bone collagen [21–27] with error bars showing 1 s.d. (*b*) Proportions of fauna bones in prehistoric assemblages determined from NISP. Owing to the over-representation of shellfish, this class has been excluded; however, shellfish comprise just 1.3% of Neolithic assemblages. Faunal skeletal assemblages from the Mesolithic from this region are sparse, and comparable published datasets almost non-existent; the Mesolithic bar here therefore represents the faunal data from a single site of Northton, Isle of Harris [28]. Although no marine mammals were recorded at this site, a high number were recorded at Oronsay [29]. (*c*) The percentage of pot lipid residues containing components diagnostic of aquatic fat processing and (*d*) the percentage of pot residues that were classified as dairy fats.

Such low prevalence of marine biomarkers in these pottery residues, less than 1% from over 40 sites, is inconsistent with significant exploitation of aquatic resources throughout the Neolithic. Moreover, the composition of organic residues from the post-Neolithic pottery demonstrates that biomarkers of aquatic origin remain rare over the subsequent 2 millennia. No evidence for marine product processing, based on long-chain DHYAs and APAAs, was identified from 40 sherds of Bronze Age pottery, and marine biomarkers and more enriched stable carbon isotope signatures only re-emerge by the Late Iron Age (figure 1*d,e*). By the Viking and Norse period, from which nearly 100 sherds were investigated, marine biomarkers are considerably more widely detected in pottery (approx. 40% sherds; figures 1–3).

### (b) Dairy product processing

While aquatic commodities were rarely identified in the lipid residues of Neolithic pottery of the northeast Atlantic archipelagos, the intact triacylglycerols often exhibited a wide acyl carbon number distribution. This distribution is characteristic of dairy-derived fats, arising from the higher abundance of lower carbon number (C_12_–C_14_) fatty acids in milk fats compared with adipose fats. The stable carbon isotope signatures confirmed an overwhelming predominance of dairy products associated with Neolithic pottery throughout the northeast archipelago (figure 1*a–c,g–i*). During the earliest Neolithic, dairy fats comprised some 80% of lipid residues identified in pottery vessels (figures 1 and 3) and thus strong evidence now associates the introduction of the earliest pottery with the exploitation of secondary (liquid) animal products. At more northerly and westerly locations in the northeast Atlantic archipelago, this pattern was maintained into the Late Neolithic; however, on mainland England and Scotland the prevalence of dairy fats dropped by the later part of the third millennium BC, with terrestrial carcass products starting to play a more predominant role (figures 1*i* and 3*d*). Contributions from wild terrestrial fauna (notably deer) can be discounted, since they typically make up less than 10% of faunal assemblages on the Western Isles of Scotland [30] and are sparse on Ireland (1–3%) until the Later Neolithic [31].

### (c) Environmental effects

The non-parametric Mann–Whitney *U*-test (SPSS v. 21) was run to determine whether there was any significant difference between the δ^13^C values of Neolithic milk (Δ^13^C ≤ −3.4‰) fatty acids from island compared with mainland coastal locations. Analysis of milk residues only was chosen in order to remove any possibility of different types of fat affecting the analysis. Distributions of δ^13^C values from island and mainland locations were similar, as assessed by visual inspection, for both C_16:0_ and C_18:0_ fatty acids, and the dataset did not deviate significantly from the assumptions required for a Mann–Whitney *U*-test. Interestingly, an offset is observed between the range of compound-specific δ^13^C values from the smaller and larger island masses. The median δ^13^C value was statistically significantly higher for both fatty acids from island locations (*n*_1_ = 129, C_16:0_ = −27.0‰, C_18:0_ = −32.0‰) compared with mainland locations (*n*_2_ = 67, C_16:0_ = −28.1‰, C_18:0_ = −33.3‰; C_16:0_ *U* = 1454.5, *z* = −7.612, *p* < 0.001; C_18:0_ *U* = 1446.5, *z* = −7.633, *p* < 0.001).

These differences between the absolute values of fatty acids from milk fats can probably be explained by the greater relative expanse of coastline. More saline conditions, which are encountered at shoreline locations, are known to cause stomatal closure and hence higher stable carbon isotope signatures in the terrestrial non-halophytic plants [32,33]. This would be incorporated into the tissues of herbivore consumers, thus explaining the higher δ^13^C signatures in the herbivore fats in Hebridean, Shetlandic and Orcadian pottery.

### (d) Intercomparison of pottery lipid, human stable isotope and faunal proxies

Comparing these lipid biomarker proxies with our synthesis of faunal and human skeletal stable carbon isotope data from the northern island and coastal sites further confirms that marine products were of low importance during the Neolithic compared with those of terrestrial origin (figure 3). Indeed, significant proportions of aquatic biomarkers and more positive δ^13^C values are only observed in Late Iron Age and Viking/Norse residues. When viewed together with the archaeozoological evidence and human collagen δ^13^C values from these later periods (figures 1*d–f*, 2 and 3), our findings reveal that the immediate shift in subsistence patterns in the earliest Neolithic was followed by a gradual return to the inclusion of marine products over subsequent millennia, reaching its highest levels by the Late Norse period. By this point, greater regional variability in resource exploitation is also observed, with a strong emphasis on processing of aquatic resources detected on Shetland, where it is likely that intensive, possibly commercial fishing was undertaken [34].

## 4. Conclusion

To summarize, our findings provide unequivocal evidence that marine products were of little overall importance to the Neolithic farmers of the northeast Atlantic archipelagos, as evidenced by (i) almost non-existent evidence for aquatic product processing in pottery, (ii) low presence of aquatic species in faunal assemblages from southern Britain [35] and the Western and Northern Isles of Scotland (figure 3*b*), and (iii) collagen stable carbon isotope signatures of Mesolithic and Neolithic humans from Britain showing a terrestrial-based diet of coastal-dwellers in the Neolithic (figure 3*a*) [4,5,21]. While interpretations based on the stable carbon isotope values have been challenged, our new evidence for a widespread intensive dairy economy across the region provides an entirely plausible explanation for the high terrestrial carbon isotope signal recorded in human collagen and seemingly negates any arguments for these farmers consuming low-protein diets that would be required to mask a marine dietary contribution. Interestingly, stable carbon isotope evidence from Middle Neolithic humans from the Channel Isles [36] and our investigations of Early/Early–Mid Neolithic pottery from Jersey, which are significantly earlier and related to a northwest French Neolithic tradition (table 1), produce much the same picture to the northerly sites.

This contrasts markedly with recent findings from Late Mesolithic and Neolithic human bone stable isotopes and organic residues from pottery from the Baltic region [37–39], which indicate significant continuation of hunter–fisher–forager activities alongside the adoption of domesticates. Considering the broader impact of our findings, it should be emphasized that: (i) the contrasting Neolithization trajectories occurring contemporaneously in different regions (e.g. northeast Atlantic archipelagos versus the Baltic) point to geographically distinct ecological, demographic and cultural influences dictating the patterns of adoption of subsistence practices at this key phase of European prehistory; (ii) the rapid shift to an intensive dairy economy, persisting for several millennia, is consistent with current theories concerning the high abundance of lactase persistence among the modern inhabitants of northwest European archipelagos, which are predicated on high milk consumption driving the evolution of the -13910*T allele, and would have been enhanced by the practices introduced by the milk-producing dairy farming populations identified herein [40–43]; and (iii) these findings are relevant to the debated calcium absorption hypothesis, which emphasizes milk consumption to be of particular importance for high latitude populations where low UV light exposure can result in vitamin D deficiency and thus poor calcium absorption [44]. Given the absence of any indication of the consumption of vitamin D-rich marine resources, the suggested calcium absorption-stimulating effect of milk consumption may have been critical in maintaining the fitness of these prehistoric farming populations. Notwithstanding the need for further work to confirm the genetic characteristics and bone health of the early dairying populations of the northwest European archipelago, their continued success exploiting such marginal zones is indisputable.

## Supplementary Material

Supplementary information to: Immediate replacement of fishing with dairying by the earliest farmers of the NE Atlantic archipelagos

## Data accessibility

— Archaeological GC and GC/MS data: Bristol University Research Data Repository (doi:10.5523/bris.13kidnrls4jnl1m806eyfd8h6z).
— Archaeological single compound stable isotope data: Bristol University Research Data Repository (doi:10.5523/bris.upjtf9os1dzr154phmgvrupib).
