# Supplementary material for: Immediate replacement of fishing with dairying by the earliest farmers of the northeast Atlantic archipelagos
Source: Proc Biol Sci. 2014 Apr 7;281(1780):20132372. doi: 10.1098/rspb.2013.2372 (PMC4027381; doi:10.1098/rspb.2013.2372)
Supplement: Supplementary information to: Immediate replacement of fishing with dairying by the earliest farmers of the NE Atlantic archipelagos [file rspb20132372supp1.pdf]

Supplementary materials to:

Immediate replacement of fishing with dairying by the earliest farmers of the NE Atlantic archipelagos

Lucy J E Cramp, Jennifer Jones, Alison Sheridan, Jessica Smyth, Helen Whelton, Jacqui Mulville, Niall Sharples & Richard P Evershed

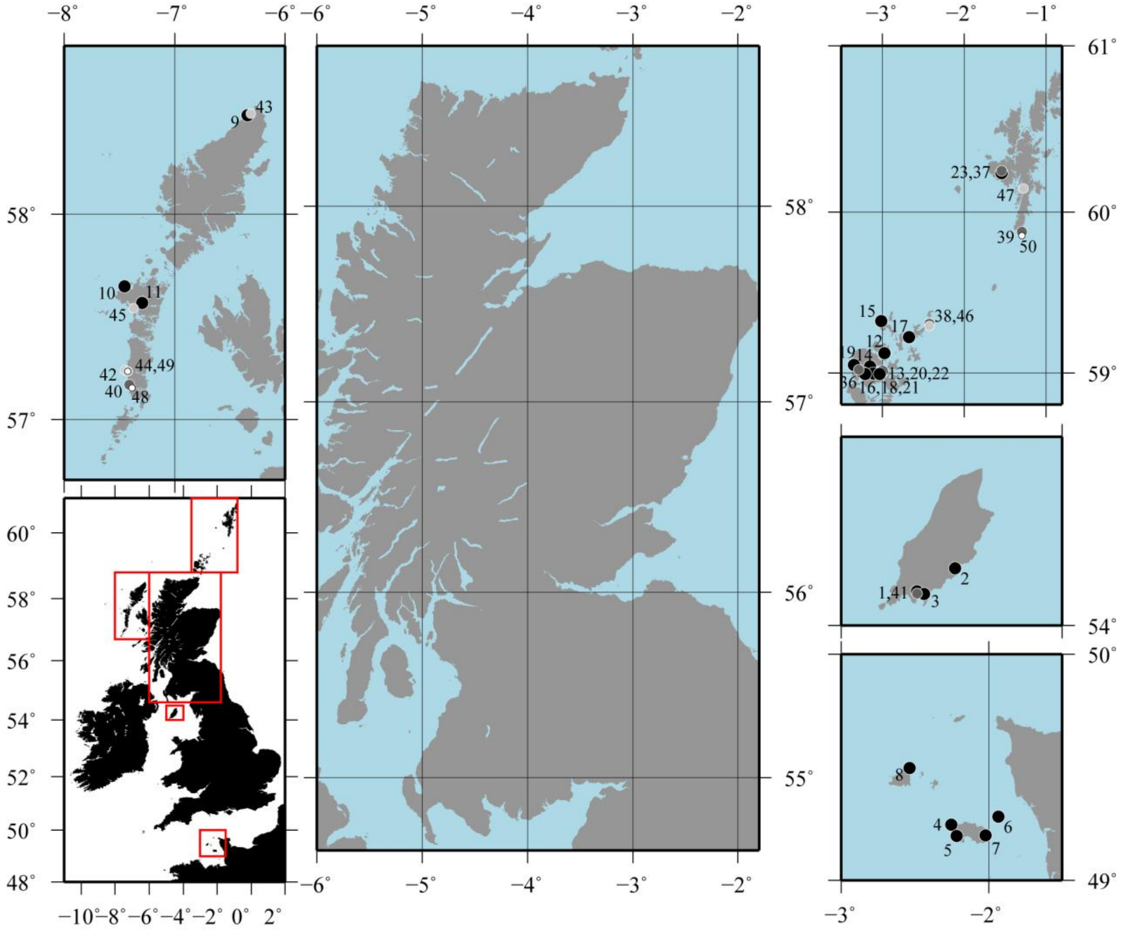

Key

Neolithic
1 Billown
2 Glencrutchery
3 Ronaldsway
4 Le Pinacle
5 Les Blanches Banques
6 Les Ecrehous
7 Mont Orgueil
8 Les Fouillages
9 Dunasbroc
10 Loch Olabhat
11 Bharpa Langais
12 Braes o'Habreck
13 Crossiecrown
14 Knowes of Trotty
15 Links of Noltland
16 Ness of Brodgar
17 Pool
18 Quanterness
19 Skara Brae
20 Stonehall
21 Stones of Stenness
22 Wideford Hill
23 Scord of Brouster
24 Culduthel
25 Doon Hill
26 Girvan Warehouse
27 Knocknab
28 Lesmurdie Road, Elgin
29 Lockerbie
30 Maybole
31 Mountcastle Quarry
32 Powmyre Quarry
33 The Hirsel
34 Upper Forth Crossing
35 Lanton Quarry

Bronze Age
36 Linga Fiold
37 Scord of Brouster
38 Toft's Ness
39 Sumburgh
40 Cladh Hallan
41 Billown

Iron Age
42 Dun Vulan
43 Dun Arnistean
44 Bornais
45 Baile Sear
46 Toft's Ness
47 Scalloway

Viking/Norse
48 Cille Pheadair
49 Bornais
50 Jarlshof

Supplementary figure S1. Map showing location of sites in northern Britain, the Channel Isles and the Isle of Man.

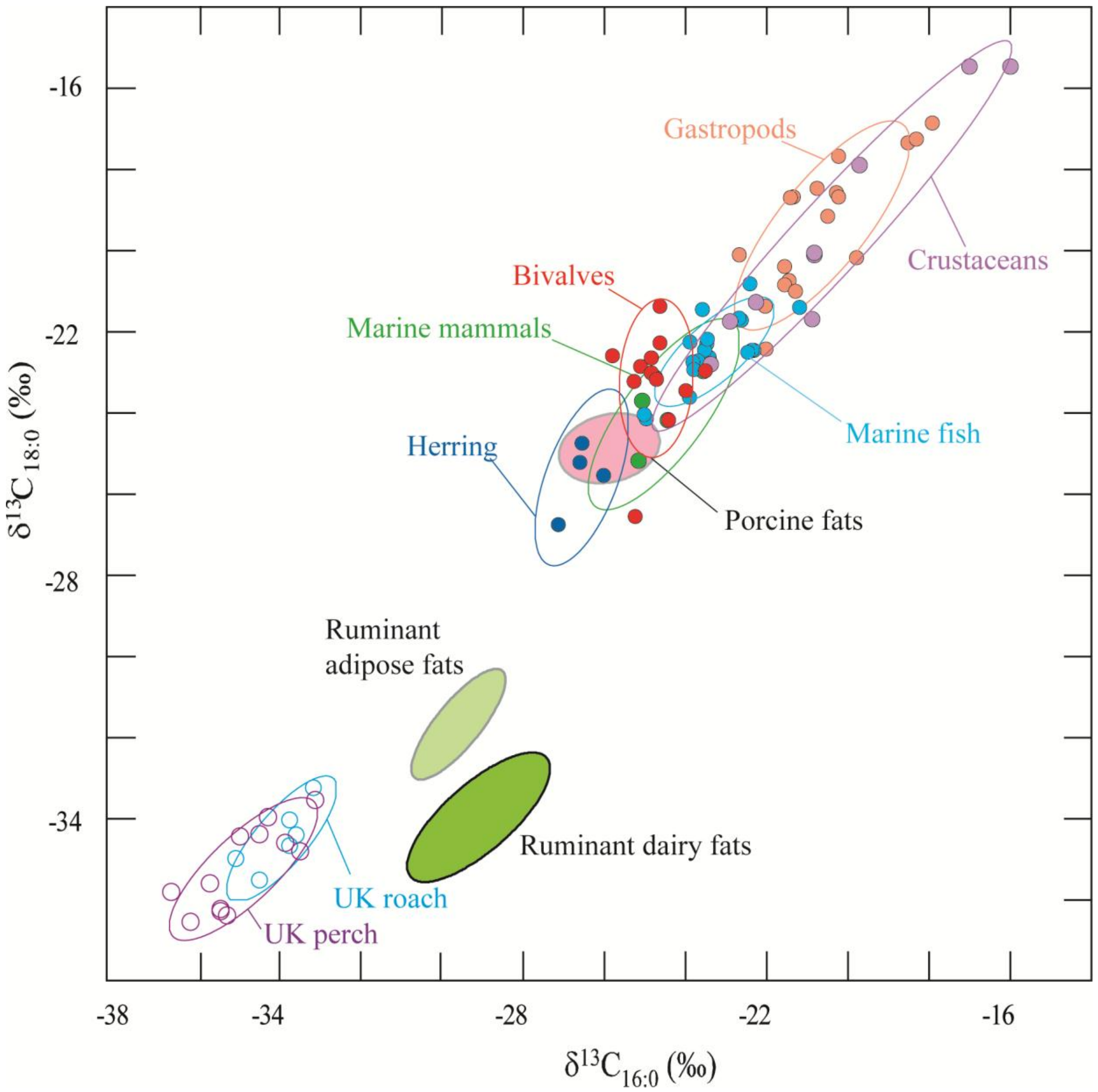

Supplementary figure S2. Plot of $\delta^{13}C$ values of individual *n*-alkanoic acids from various terrestrial and aquatic fauna collected from the UK shown with 1 s.d. confidence ellipses [1]. Reference fats were either hand-collected or sourced *via* fish merchants from a range of unfarmed locations, including the English Channel, the Severn Estuary and the North Sea. Specimens were frozen until required. Samples of tissue were removed and freeze-dried to constant weight prior to lipid extraction using 3 x 10 ml $CHCl_3$/MeOH (2:1 *v/v*). Approximately 2 mg lipid was then hydrolysed and methylated prior to analysis using GC, G/MS and GC/C/IRMS as described above. Modern reference fats were corrected for the effects of post-industrial carbon (+ 1.3 ‰; [2]).

References

(1) Cramp LJE, Evershed RP. 2014 Reconstructing Aquatic Resource Exploitation in Human Prehistory using Lipid Biomarkers and Stable Isotopes. In *Treatise on Geochemistry 2nd edition, Vol. 14* (eds. HD Holland, KK Turekian), pp. 319 – 339. Oxford: Elsevier.

(2) Friedli H, Lotscher H, Oeschger H, Siegenthaler U, Stauffer B. 1986 Ice Core Record of the C-13/C-12 Ratio of atmospheric CO_2 in the past two centuries. *Nature* **324**, 237-238.

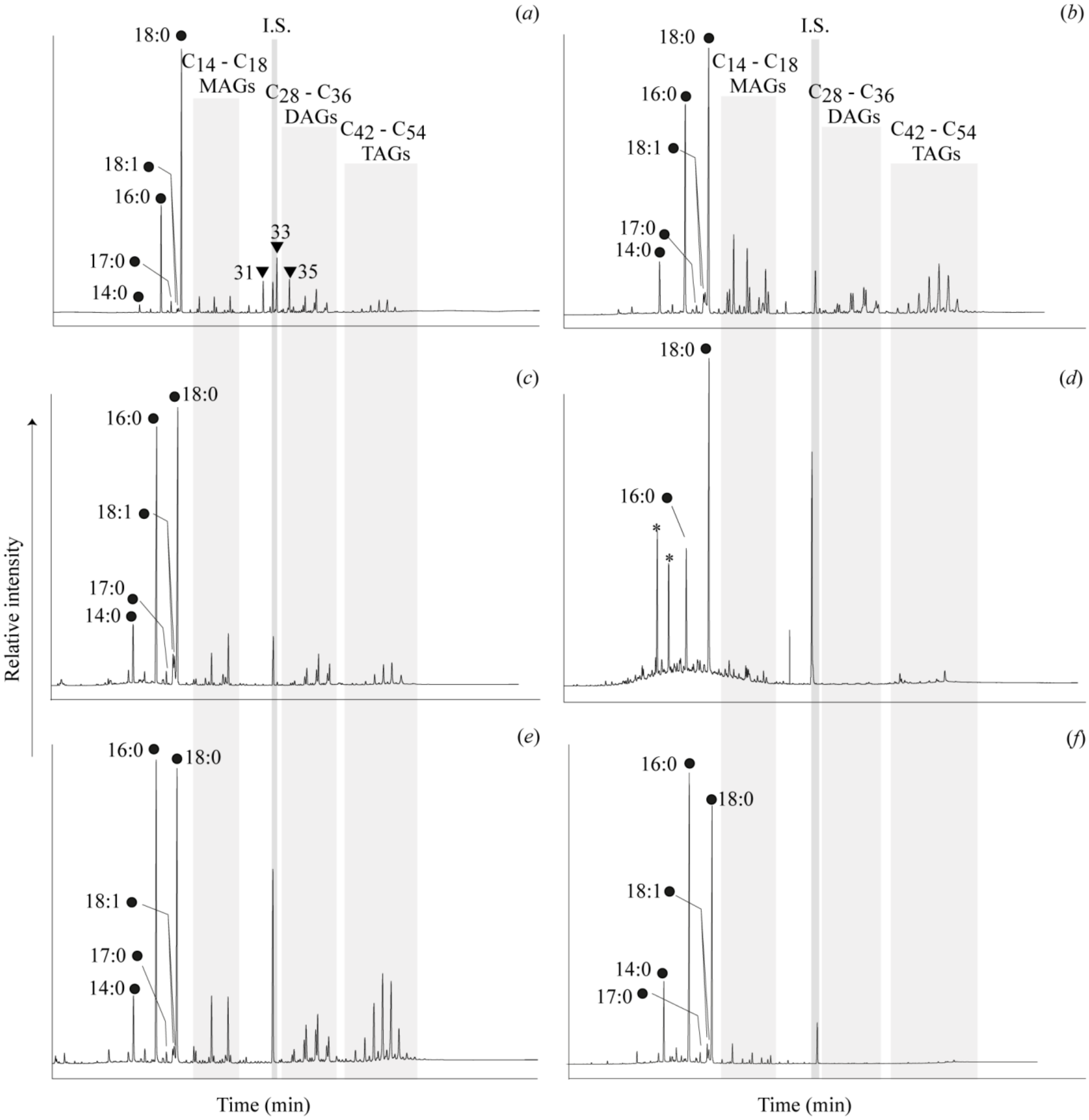

Supplementary figure S3. Partial high temperature gas chromatograms from organic residues from Neolithic pot sherds from (*a*) Culduthel, Invernesshire, (*b*) Dunasbroc, Isle of Lewis, (*c*) Billown, Isle of Man, (*d*) Les Fouaillages, Guernsey, (*e*) Braes o'Habreck, Wyre and (*f*) Scord of Brouster, Shetland. Filled circles – free fatty acid with *x* carbon atoms and *y* degree of unsaturation, *filled triangles* – mid-chain ketone with *x* carbon atoms, MAGs – monoacylglycerols, DAGs – diacylglycerols, TAGs – triacylglycerols, I.S. – internal standard (C_{34} *n*-alkane). Time retention windows are from 5 - 42 min.

Supplementary table S1. Table showing fragment ions selected for GC/MS-SIM for detection of C_{16} – C_{22} APAAs.

	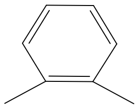	
**Chain length**	**Base peak (*m/z*)**	**$M^{+\cdot}$ (*m/z*)**
16	105	262
18	105	290
20	105	318
22	105	346

Supplementary table S2. Major fragment and $[M-15]^+$ ions used for GC/MS-SIM of the vicinal diols with carbon chain lengths of 18 to 22. The fragment ions of the major positional isomers are highlighted in grey.

	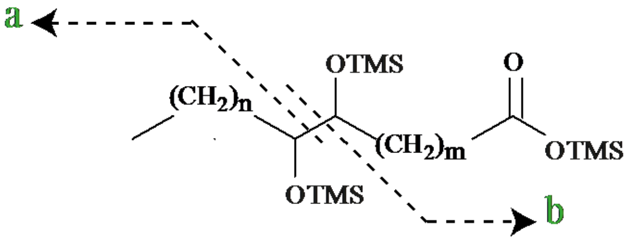		
Dihydroxy acid	a	b	$[M – 15]^+$
	m/z		
9,10-C_{18}	215	317	517
11,12-C_{18}	187	345	517
13,14-C_{18}	159	373	517
9,10-C_{20}	243	317	545
11,12-C_{20}	215	345	545
11,12-C_{22}	243	345	573
13,14-C_{22}	215	373	573

Supplementary table S3. List of sherds from coastal sites investigated for aquatic biomarkers

Sherd	$\delta^{13}C_{16:0}$	$\delta^{13}C_{18:0}$	$\Delta^{13}C$	Aquatic biomarkers
CHANNEL ISLES/NORMANDY TRADITION NEOLITHIC				
EARLY NEOLITHIC	*Villeneuve-Saint-Germain, Pinacle-Fouillages*			
**Les Fouillages**, Guernsey				
LF1	-29.8	-33.4	-3.5	(-)
LF4	-28.3	-33.5	-5.2	(-)
LF5	-27.0	-31.3	-4.3	(-)
EARLY MID NEOLITHIC	*Cerny- and early Castellic-type*			
**Les Blanches Banques,** Jersey				
LBB1	-26.8	-29.6	-2.8	(-)
LBB2	-27.9	-31.3	-3.4	(-)
**Mont Orgueil**, Jersey				
MOG1	-27.4	-32.6	-5.2	(-)
MOG3	-27.9	-31.2	-3.3	(-)
MOG4	-27.9	-32.9	-5.1	(-)
NEOLITHIC BRITAIN AND ISLE OF MAN				
EARLY NEOLITHIC	*Carinated Bowl, modified Carinated Bowl, Mull Hill ware*			
**Mainland Northern Britain**				
**Maybole,** Ayrshire				
MB3	-26.9	-30.4	-3.5	(-)
MB4	-28.3	-34.2	-5.9	(-)
MB5	-27.6	-32.7	-5.2	(-)
MB6	-29.2	-33.6	-4.4	(-)
**Lockerbie,** Dumfries & Galloway				
LK1	-27.8	-33.1	-5.3	(-)
LK3	-28.5	-33.2	-4.7	(-)
**Doon Hill** East Lothian				
DH1	-27.3	-32.7	-5.4	(-)

DH4	-27.2	-33.3	-6.2	(-)
**Lesmurdie Road,** Elgin, Moray				
LR1	-27.6	-29.1	-1.6	LC APAAs, LC DHYAs
**The Hirsel,** Roxburghshire				
HIR1	-28.6	-32.2	-3.6	(-)
HS1	-27.6	-30.9	-3.3	(-)
HS2	-27.0	-32.9	-5.8	(-)
**Girvan Warehouse,** Ayrshire				
GIR1	-29.0	-32.8	-3.8	(-)
GIR3	-27.7	-32.5	-4.8	(-)
GIR4	-29.7	-34.6	-4.9	(-)
GIR5	-29.3	-35.7	-6.4	(-)
GIR7	-28.4	-34.1	-5.6	(-)
GIR11M	-28.8	-33.0	-4.2	(-)
GIR11R	-31.0	-32.7	-1.7	(-)
**Knocknab,** Luce Sands				
KN1	-26.4	-32.0	-5.6	(-)
KN3	-24.6	-26.8	-2.2	(-)
**Lanton Quarry,** Northumberland				
LAN1	-28.7	-34.2	-5.5	(-)
LAN2	-28.1	-32.6	-4.6	(-)
LAN3	-27.2	-30.1	-2.9	(-)
LAN4	-28.1	-30.4	-2.3	(-)
LAN5	-28.0	-34.1	-6.1	(-)
LAN6R	-28.1	-33.2	-5.1	(-)
LAN10V1	-28.1	-33.4	-5.4	(-)
LAN10V2	-29.7	-32.8	-3.1	(-)
LAN15	-28.1	-32.4	-4.3	(-)
LAN16	-27.6	-33.2	-5.6	(-)
LAN20	-28.2	-32.9	-4.8	(-)
LAN22	-27.5	-33.2	-5.7	(-)
LAN23	-27.8	-33.4	-5.6	(-)
LAN25	-27.0	-32.9	-5.9	(-)
LAN28R	-27.4	-31.8	-4.4	(-)
LAN28M	-28.9	-31.6	-2.7	(-)
LAN29	-27.3	-33.2	-5.9	(-)
LAN30	-27.4	-32.7	-5.4	(-)
LAN31	-27.8	-33.5	-5.7	(-)
LAN33	-28.5	-32.7	-4.2	(-)
LAN39	-28.1	-31.8	-3.8	(-)
LAN40M	-27.7	-31.7	-4.0	(-)
LAN42	-28.4	-33.3	-4.9	(-)
LAN43R	-28.0	-33.4	-5.5	(-)

LAN43M	-27.7	-33.1	-5.4	(-)
LAN44	-27.3	-31.2	-3.9	(-)
LAN45	-26.5	-32.8	-6.3	(-)
LAN46	-27.4	-33.0	-5.6	(-)
LAN47	-28.5	-32.7	-4.1	(-)
LAN49	-28.3	-32.8	-4.5	(-)
LAN50	-28.1	-29.9	-1.8	(-)
LAN51	-25.8	-31.9	-6.1	(-)
**Upper Forth Crossing,** East Lothian				
UFC4	-27.6	-31.7	-4.2	(-)
**Isle of Man**				
**Billown**				
BIL1	-30.2	-35.4	-5.2	(-)
BIL2	-27.8	-34.3	-6.4	(-)
BIL6	-28.1	-32.1	-4.0	(-)
BIL8	-28.0	-31.6	-3.7	(-)
BIL10	-28.9	-34.3	-5.5	(-)
BIL11	-28.6	-33.7	-5.0	(-)
BIL12	-28.3	-31.5	-3.2	(-)
BIL15	-28.8	-31.7	-2.9	(-)
BIL20	-27.1	-28.8	-1.7	(-)
BIL20V	-27.5	-30.9	-3.4	(-)
SECONDARY EXPANSION	*Hebridean, 'Unstan', Impressed, Early Grooved Wares*			
**Mainland Northern Britain**				
**Culduthel**, Invernesshire				
CUL1	-27.5	-35.0	-7.5	(-)
CUL4	-28.7	-34.0	-5.3	(-)
CUL4V	-30.9	-37.2	-6.3	(-)
**Upper Forth Crossing,** Clackmannanshire				
UFC7	-28.7	-34.7	-5.9	(-)
UFC8	-30.3	-35.4	-5.0	(-)
UFC11	-27.0	-33.7	-6.7	(-)
UFC12	-29.9	-34.8	-4.9	(-)
UFC15	-27.4	-33.7	-6.3	(-)
UFC17	-29.2	-34.5	-5.4	(-)
UFC19	-30.6	-33.3	-2.7	(-)
UFC20V	-30.6	-33.2	-2.7	(-)
UFC21	-28.0	-33.9	-5.9	(-)
UFC22	-29.2	-34.1	-4.8	(-)
UFC22V	-29.3	-33.6	-4.3	(-)

UFC23	-28.9	-34.5	-5.7	(-)
UFC24	-30.0	-35.5	-5.5	(-)
UFC25	-29.6	-33.9	-4.3	(-)
UFC27	-30.2	-35.6	-5.4	(-)
**Outer Hebrides**				
**Dunasbroc**, Isle of Lewis				
DB-2	-26.0	-33.2	-7.1	(-)
DB-7	-27.1	-29.4	-2.3	(-)
DB-8	-27.3	-32.6	-5.3	(-)
DB-9	-27.2	-32.4	-5.2	(-)
DB-11	-27.5	-30.8	-3.3	(-)
DB-12	-27.4	-32.3	-4.9	(-)
DB-13	-26.5	-31.8	-5.4	(-)
DB-14	-27.4	-29.8	-2.4	(-)
DB-15	-26.5	-32.8	-6.3	(-)
DB-16	-24.4	-32.8	-8.5	(-)
DB-17	-27.2	-32.9	-5.7	(-)
DB-20	-27.2	-32.6	-5.4	(-)
DB-22	-26.1	-32.9	-6.8	(-)
DB-24	-26.5	-32.8	-6.3	(-)
DB-25	-25.7	-32.5	-6.8	(-)
DB-26	-26.5	-32.1	-5.6	(-)
DB-28	-26.1	-30.4	-4.3	(-)
DB-30	-26.6	-31.7	-5.1	(-)
**Loch Olabhat,** North Uist				
LOL2	-26.0	-33.2	-7.1	(-)
LOL4	-27.1	-29.4	-2.3	(-)
LOL5	-27.3	-32.6	-5.3	(-)
LOL8	-27.2	-32.4	-5.2	(-)
LOL14	-27.5	-30.8	-3.3	(-)
LOL14V	-27.4	-32.3	-4.9	(-)
LOL23	-26.5	-31.8	-5.4	(-)
LOL25	-27.4	-29.8	-2.4	(-)
**Bharpa Langais,** South Uist				
BP1	-26.6	-32.1	-5.5	LC APAAs, LC DHYAs
BP1V	-28.2	-30.5	-2.4	-
**Orkney**				
**Pool,** Sanday				
PL2	-27.6	-31.1	-3.5	(-)
**Braes o'Habreck**, Wyre				
HAB6	-28.3	-31.5	-3.1	(-)
HAB7	n.a.	n.a.	n.a.	(-)

HAB9	n.a.	n.a.	n.a.	(-)
HAB15	-27.0	-33.4	-6.4	(-)
HAB17	-27.8	-30.7	-3.0	(-)
**Knowes of Trotty,** Mainland				
KOT2	-28.4	-31.6	-3.2	(-)
KOT4	-27.6	-30.7	-3.2	(-)
KOT5	-27.1	-31.0	-3.9	(-)
KOT6	-26.3	-26.6	-0.3	(-)
KOT7	-27.0	-32.6	-5.6	(-)
KOT8	-28.3	-33.1	-4.8	(-)
KOT10	-26.6	-31.4	-4.7	(-)
**Stonehall,** Mainland[†]				
SHU29	-28.5	-33.6	-5.1	(-)
SHU168	-27.4	-31.1	-3.7	(-)
SHR168	-27.5	-31.1	-3.6	(-)
SHU1230	-28.0	-31.9	-3.9	(-)
SHU7118	-27.5	-32.8	-5.3	(-)
SHU7119	-27.5	-31.8	-4.3	(-)
SHU7055	-28.3	-33.3	-5.0	(-)
SHR7055	-28.5	-33.5	-5.0	(-)
SHU7064	-27.7	-32.6	-4.9	n.a.
SHU7094	-27.5	-32.0	-4.5	(-)
SHU7145	-28.7	-32.1	-3.4	n.a.
SH1800	-24.6	-31.1	-6.5	n.a.
SH2300	-27.8	-30.3	-2.5	n.a.
SH2430	-27.5	-29.9	-2.4	(-)
SH2499	-28.4	-32.6	-4.2	(-)
**Wideford Hill,** Mainland[†]				
WHU6	-26.8	-32.8	-6.0	(-)
WHU13	-26.4	-32.9	-6.5	(-)
WHU286	-27.4	-32.9	-5.5	(-)
WHU290	-27.9	-33.3	-5.4	(-)
WHU399	-26.6	-31.0	-4.4	(-)
WHU567	-28.4	-33.8	-5.4	(-)
WHU636	-26.9	-32.9	-6.0	(-)
WHR636	-28.3	-33.1	-4.8	(-)
WHU912	-26.8	-32.6	-5.8	n.a.
WHU915	-27.2	-33.0	-5.8	n.a.
WHU995	-26.9	-30.7	-3.8	(-)
**Quanterness,** Mainland				
QAN2	-28.9	-33.5	-4.6	(-)
QAN3	-28.3	-31.9	-3.6	(-)
QAN4	-28.9	-32.8	-3.9	(-)
QAN5	-27.1	-33.1	-5.9	(-)
**Shetland**				

**Scord of Brouster**				
SOB6	n.a.	n.a.	n.a.	(-)
LATE NEOLITHIC		*Grooved Ware, Ronaldsway Ware*		
**Mainland Northern Britain**				
**Mountcastle Quarry**				
MQLF2	-27.7	-29.8	-2.0	(-)
MQLF3	-28.9	-29.8	-0.9	(-)
**Orkney**				
**Pool,** Sanday				
PL1	n.a.	n.a.	n.a.	(-)
PL3	-25.8	-29.7	-3.8	(-)
PL5	-28.7	-32.0	-3.4	(-)
PL7	-27.8	-33.2	-5.4	(-)
**Ness of Brodgar,** Mainland				
NEB1	-26.8	-29.1	-2.3	(-)
NEB2	-26.7	-30.4	-3.6	(-)
NEB3	n.a.	n.a.	n.a.	(-)
NEB4	-27.4	-30.1	-2.7	(-)
NEB6	-27.5	-29.4	-1.9	(-)
NEB7	-27.3	-31.7	-4.5	(-)
NEB8	-27.6	-31.3	-3.7	(-)
NEB10	-27.2	-32.0	-4.8	(-)
NEB13	-28.1	-30.7	-2.6	(-)
NEB15	-27.0	-30.8	-3.8	(-)
NEB16	-26.5	-30.5	-4.0	(-)
NEB17	-27.4	-32.3	-4.8	(-)
NEB18	-26.7	-31.0	-4.3	(-)
NEB19	-26.7	-30.0	-3.3	(-)
NEB21	-26.6	-29.0	-2.5	(-)
NEB22	n.a.	n.a.	n.a.	(-)
NEB23	-27.6	-30.9	-3.3	(-)
NEB25	-30.0	-29.1	0.8	(-)
NEB26	-26.6	-29.4	-2.8	(-)
NEB27	-26.4	-31.0	-4.6	(-)
NEB28	-28.1	-31.4	-3.4	(-)
NEB30	-27.3	-30.3	-3.0	(-)
**Skara Brae,** Mainland†				
SBG27	-27.2	-29.2	-2.0	(-)
SBG40	-25.0	-29.9	-4.9	n.a.
SBG84	-27.4	-30.4	-3.0	n.a.
SBG97	-28.0	-30.8	-2.8	(-)
SBG104	-26.8	-30.0	-3.2	(-)

SBG234	-27.7	-31.1	-3.4	(-)
SBR234	-27.0	-31.5	-4.5	n.a.
SBG240	-27.1	-27.1	0.0	n.a.
SBG317	-27.8	-30.5	-2.7	(-)
SBG332	-27.0	-29.8	-2.8	n.a.
SBG338	-27.4	-30.5	-3.1	(-)
SBG340	-27.0	-30.9	-3.9	(-)
SBG349	-26.3	-32.9	-6.6	(-)
SBG385	-27.8	-31.5	-3.7	(-)
SBG507	-25.6	-30.1	-4.5	(-)
SBG761	-27.6	-31.1	-3.5	(-)
SBG801	-27.3	-32.2	-4.9	n.a.
SBG1000	-27.9	-32.5	-4.6	(-)
SBG1051	-26.8	-30.4	-3.6	(-)
SBG1054	-26.5	-30.9	-4.4	(-)
SBG1055	-27.1	-30.9	-3.8	(-)
SBG1057	-25.9	-32.2	-6.3	(-)
SBG1542	-26.4	-31.5	-5.1	(-)
SBG1602	-27.8	-30.7	-2.9	(-)
SBG1639	-27.5	-31.8	-4.3	(-)
SBG1754	-27.4	-30.8	-3.4	(-)
SBG3010	-26.0	-29.7	-3.7	(-)
SBG3026	-31.8	-30.2	1.6	(-)
SBG3111	-26.4	-29.4	-3.0	(-)
SBG3137	-27.8	-30.8	-3.0	n.a.
SBG3142	-27.1	-30.7	-3.6	(-)
SBG3191	-27.3	-30.6	-3.3	(-)
SBG3193	-27.5	-30.4	-2.9	n.a.
SBG3198	-27.1	-29.9	-2.8	(-)
SBG3241	-27.8	-30.7	-2.9	(-)
SBG5149	-27.4	-30.6	-3.2	(-)
**Stonehall,** Mainland[†]				
SHG393	-27.6	-31.6	-4.0	n.a.
SHG2106	-26.8	-32.0	-5.2	
SHG2107	-28.2	-31.6	-3.4	
SHG2134	-27.9	-28.6	-0.7	(-)
SHG2150	-27.4	-30.0	-2.6	(-)
SHG2625	-27.9	-32.1	-4.2	n.a.
SHG3606	-28.4	-31.6	-3.2	(-)
SHG4124	-27.7	-30.7	-3.0	(-)
SHG6077	-27.2	-31.8	-4.6	n.a.
SHG6260	-26.8	-33.6	-6.8	n.a.
SHG6261	-27.2	-30.0	-2.8	(-)
SHG6306	-27.1	-30.4	-3.3	(-)
SHR6306	-27.0	-30.3	-3.3	(-)
SHR6305	-27.1	-32.0	-4.9	(-)
**Crossiecrown,** Mainland[†]				
CCG19	-28.1	-33.7	-5.6	(-)

CCG150	-26.6	-32.4	-5.8	(-)
CCG738	-29.6	-31.9	-2.3	(-)
CCG899	-28.0	-31.7	-3.7	(-)
CCG937	-28.1	-30.2	-2.1	n.a.
**Links of Noltland,** Westray†				
LON1	-26.4	-30.2	-3.8	(-)
LON2	-25.3	-32.0	-6.8	(-)
LON3	-24.9	-27.8	-2.9	(-)
LON4	-26.2	-32.3	-6.1	(-)
LON5	-26.0	-30.5	-4.5	(-)
LON7	-27.0	-29.7	-2.7	(-)
LON10	-26.4	-32.5	-6.1	(-)
LON11	-27.4	-29.9	-2.6	(-)
LON12	-26.1	-31.2	-5.0	(-)
LON13	-25.5	-29.5	-4.0	(-)
LON14	-25.8	-32.1	-6.3	(-)
LON16	-25.1	-28.5	-3.3	(-)
LON18	-25.5	-31.1	-5.6	(-)
LON19	-26.1	-29.3	-3.2	(-)
LON23	-26.7	-28.8	-2.1	(-)
LON24	-25.8	-31.7	-5.9	(-)
LON30	-27.6	-30.5	-2.9	(-)
LON31	-24.2	-30.7	-6.5	(-)
LON32	-27.3	-32.0	-4.7	(-)
LON33	-25.8	-32.4	-6.6	(-)
LON34	-26.0	-32.0	-6.0	(-)
LON35	-27.9	-30.0	-2.1	(-)
LON36	-28.5	-30.0	-1.5	(-)
LON38	-28.7	-30.5	-1.8	(-)
LON39	-27.4	-29.4	-2.0	(-)
LON40	-26.7	-30.2	-3.5	(-)
LON40V	-26.1	-29.4	-3.3	(-)
LON41	-26.4	-29.3	-2.9	(-)
LON42	-26.9	-29.7	-2.8	(-)
LON43	-28.6	-33.7	-5.1	(-)
LON44V	-26.6	-28.8	-2.3	(-)
LON46	-26.1	-29.6	-3.5	(-)
LON46V	-27.3	-30.5	-3.3	(-)
LON50	-26.8	-29.6	-2.7	(-)
LON52	-27.7	-30.6	-2.9	(-)
LON52V	-26.6	-29.6	-3.1	(-)
LNG1	-26.4	-29.0	-2.6	n.a.
LNG2	-25.7	-31.5	-5.8	n.a.
LNG3	-27.1	-30.3	-3.2	n.a.
LNG4	-26.7	-33.4	-6.7	n.a.
LNG6	-27.2	-30.1	-2.9	n.a.
LNG7	-26.5	-28.9	-2.4	n.a.
LNG8	-25.3	-27.0	-1.7	n.a.
LNG9	-26.5	-29.0	-2.5	n.a.

LNG10	-26.6	-30.7	-4.1	n.a.
LNG11	-27.0	-31.1	-4.1	n.a.
LNG12	-27.1	-31.9	-4.8	n.a.
LNG14	-26.3	-32.3	-6.0	n.a.
LNG15	-26.0	-31.8	-5.8	n.a.
**Shetland**				
**Scord of Brouster,** Mainland				
SOB1	-27.0	-33.6	-6.6	(-)
SOB3	-27.6	-32.9	-5.3	(-)
SOB4	-27.7	-33.7	-6.1	(-)
SOB9	-27.5	-31.6	-5.9	(-)
SOB10	-27.2	-31.5	-4.2	(-)
SOB11	-27.0	-30.4	-4.4	(-)
SOB14	-27.6	-32.6	-3.4	(-)
SOB15	-28.4	-32.6	-5.0	(-)
SOB16	-28.3	-33.1	-4.1	(-)
SOB17	-27.4	-31.8	-4.8	(-)
SOB18	-27.8	-32.2	-4.4	(-)
SOB19	-27.8	-32.8	-4.4	(-)
SOB20	-27.7	-32.4	-5.1	(-)
SOB21	-28.0	-30.4	-4.7	(-)
SOB22	-27.7	-32.5	-2.4	(-)
**Isle of Man**				
**Billown**				
BIL21	-28.4	-33.3	-4.8	(-)
**Ronaldsway**				
RW1	-26.1	-31.8	-5.6	(-)
RW2	-27.1	-33.1	-6.0	(-)
RW3	-27.4	-33.1	-5.7	(-)
RW4	n.a.	n.a.	n.a.	(-)
RW7	-25.8	-27.1	-1.3	(-)
RW8	-27.4	-32.8	-5.5	(-)
RW10	-27.7	-33.9	-6.2	(-)
RW14	-28.5	-33.9	-5.4	(-)
RW17	-26.7	-32.1	-5.4	(-)
RW18	-27.3	-32.9	-5.6	(-)
RW20	-30.3	-34.8	-4.5	(-)
RW21	-28.5	-33.8	-5.3	(-)
RW24	-27.2	-32.7	-5.5	(-)
RW25	n.a.	n.a.	n.a.	(-)
RW30	-25.5	-31.9	-6.5	(-)
RW30V	-26.6	-33.1	-6.5	(-)
**Glencrutchery**				

GC5	-31.4	-35.0	-3.5	(-)
GC14	-31.4	-34.3	-2.9	(-)
GC23	-29.1	-31.9	-2.8	(-)
GC28	-29.7	-34.2	-4.5	(-)

BRONZE AGE

Outer Hebrides

Cladh Hallan, South Uist

CH2	-26.2	-28.5	-2.3	(-)
CH3	-27.7	-29.0	-1.4	(-)
CH4	-27.6	-32.6	-4.9	(-)
CH5	-27.7	-33.0	-5.3	(-)
CH6	-26.8	-32.5	-5.7	(-)
CH8	-25.9	-28.7	-2.7	(-)
CH12	-27.2	-31.8	-4.6	(-)
CH16	-27.3	-30.9	-3.6	(-)
CH17	-28.2	-31.3	-3.0	(-)

Orkney

Toft's Ness, Sanday

TN1	-27.2	-31.0	-3.9	(-)
TN4	-29.7	-32.4	-2.7	(-)
TN6	-27.9	-31.4	-3.6	(-)
TN8	-29.6	-30.8	-1.2	(-)
TN10	-29.4	-33.8	-4.4	(-)
TN11	-28.3	-32.9	-4.7	(-)
TN12	-28.2	-32.0	-3.9	(-)
TN16	-28.7	-30.1	-1.4	(-)

Linga Fiold, Mainland

LIN1	-28.2	-31.1	-2.9	(-)
LIN2	-27.8	-33.2	-5.3	(-)
LIN3	-29.0	-33.5	-4.6	(-)
LIN5	-28.3	-31.4	-3.1	(-)
LIN6	-28.0	-32.3	-4.3	(-)

Shetland

Scord of Brouster, Mainland

SOB7	-26.8	-32.7	-5.9	(-)

Sumburgh

SUM1	-26.7	-29.1	-2.4	(-)
SUM3	-25.8	-29.7	-3.9	(-)
SUM5	-26.1	-29.7	-3.6	(-)
SUM6	-26.5	-30.1	-3.6	(-)
SUM7	-27.0	-32.1	-5.1	(-)

SUM8	-26.4	-29.8	-3.4	(-)
SUM9	-27.2	-29.6	-2.4	(-)
SUM10	-26.6	-30.5	-4.0	(-)
SUM13	-26.6	-31.3	-4.6	(-)
SUM14	-26.6	-31.6	-5.0	(-)
SUM15	-26.7	-30.3	-3.5	(-)
SUM16	-26.9	-30.5	-3.5	(-)
SUM17	-26.6	-31.5	-4.8	(-)
SUM19	-26.4	-31.0	-4.6	(-)
SUM20m	-26.6	-29.5	-2.8	(-)
SUM20b	-26.0	-31.2	-5.2	(-)
SUM21	-26.5	-30.5	-4.0	(-)

Iron Age

Outer Hebrides

**Dun Vulan,** South Uist				
DUV1	-27.5	-32.8	-5.3	(-)
DUV2	-27.5	-30.8	-3.3	(-)
DUV3	-26.0	-32.1	-6.1	(-)
DUV6	-26.3	-29.6	-3.3	(-)
DUV8	-26.6	-30.4	-3.8	(-)
DUV9	-26.3	-29.9	-3.6	(-)
DUV14	-27.8	-32.6	-4.8	(-)
DUV15	-27.5	-32.0	-4.5	(-)
DUV16	-26.8	-29.2	-2.4	(-)
DUV19	-26.6	-30.5	-3.9	(-)
DUV21	-25.7	-29.9	-4.2	(-)
DUV22	-26.5	-32.1	-5.6	(-)
DUV24	-25.6	-30.8	-5.2	(-)
DUV27	-27.5	-32.5	-4.9	(-)
DUV28	-25.5	-30.8	-5.3	(-)

**Baile Sear,** North Uist				
BS1	-26.9	-30.5	-3.6	(-)
BS2	-27.2	-31.7	-4.5	(-)
BS4	-27.2	-29.0	-1.9	(-)
BS5	-26.6	-29.7	-3.2	(-)
BS6	n.a.	n.a.	n.a.	(-)
BS7	-27.0	-31.9	-4.9	(-)
BS8	-26.5	-29.5	-3.0	(-)
BS9	n.a.	n.a.	n.a.	(-)
BS10	n.a.	n.a.	n.a.	(-)
BS11	-27.1	-31.9	-4.8	(-)
BS12	n.a.	n.a.	n.a.	(-)
BS14	-26.7	-31.0	-4.4	(-)
BS15	-27.7	-30.2	-2.5	(-)
BS18	-25.8	-31.3	-5.5	(-)

BS19	-26.3	-29.1	-2.8	(-)
BS20	-27.0	-32.7	-5.7	(-)
BS21	-28.0	-32.4	-4.4	(-)
BS23	-26.4	-29.3	-2.9	(-)
BS24	-26.1	-32.5	-6.4	(-)
BS8V	-27.0	-29.6	-2.6	(-)
**Dun Arnistean,** Isle of Lewis				
DAR1	-26.7	-29.7	-3.0	(-)
DAR3	n.a.	n.a.	n.a.	(-)
DAR7	-27.3	-30.8	-3.5	(-)
DAR8	-27.7	-31.4	-3.7	(-)
DAR9	n.a.	n.a.	n.a.	(-)
**Bornais,** South Uist				
BN19	-27.0	-30.2	-3.2	(-)
BN17	-26.9	-30.8	-3.9	(-)
BN20	-27.1	-31.0	-3.9	(-)
BN25	-27.6	-32.5	-4.8	(-)
BN-26	n.a.	n.a.	n.a.	(-)
BN58	-28.3	-32.0	-3.7	(-)
BN59	n.a.	n.a.	n.a.	(-)
BN62	-27.6	-32.5	-4.8	(-)
BN66	-27.4	-30.3	-2.9	(-)
BN66v	-27.4	-30.3	-2.9	(-)
BN68	-26.8	-28.5	-1.6	(-)
BN69	-26.9	-31.0	-4.1	(-)
BN70	-26.8	-29.9	-3.1	LC APAAs
BN70V	-26.8	-29.3	-2.5	(-)
BN71	-27.4	-31.7	-4.3	(-)
BN71V	-26.9	-30.8	-3.8	(-)
BN72	-28.1	-33.8	-5.8	(-)
BN74	-26.6	-29.4	-2.8	LC APAAs
BN74V	-26.9	-29.8	-2.9	(-)
BN76	-26.8	-29.3	-2.5	(-)
BN77	-26.9	-29.9	-3.1	(-)
BN78V	-27.0	-29.7	-2.7	(-)
BN79	-26.8	-30.0	-3.2	(-)
BN80	-28.3	-32.5	-4.3	(-)
BN82	-26.8	-29.0	-2.2	(-)
BN83	-27.4	-30.6	-3.3	LC DHYAs
BN84	-28.0	-30.4	-2.4	(-)
BN86	-26.8	-29.4	-2.5	(-)
BN87	-26.8	-30.4	-3.6	LC APAAs
BN87V	-27.5	-30.7	-3.2	(-)
BN88	-24.2	-25.5	-1.3	LC APAAs, LC DHYAs
BN88V	-25.1	-28.5	-3.4	(-)
BN89	-26.8	-29.4	-2.5	(-)

**Orkney**				
**Toft's Ness,** Sanday				
TN19	-25.7	-29.4	-3.7	LC DHYAs
TN22	-26.5	-30.4	-3.9	(-)
TN25	-26.0	-28.5	-2.5	(-)
TN27	-25.8	-29.4	-3.6	(-)
TN34	-26.6	-31.0	-4.4	LC APAAs
**Shetland**				
**Scalloway,** Mainland				
SCA4v	-27.6	-27.7	-0.1	LC APAAs, LC DHYAs
SCA1	-27.6	-32.7	-5.1	(-)
SCA2	-27.8	-29.4	-1.6	LC APAAs, LC DHYAs
SCA3	-27.6	-27.7	-0.1	(-)
SCA4	-27.6	-32.7	-5.1	LC DHYAs
SCA5	-27.8	-29.4	-1.6	(-)
SCA6	-27.2	-31.1	-3.9	(-)
SCA8	-28.1	-28.1	-0.0	(-)
SCA10	-27.4	-31.6	-4.2	(-)
SCA12	-27.4	-31.3	-3.9	(-)
SCA13	-27.7	-31.9	-4.2	(-)
SCA14	-27.5	-31.3	-3.8	(-)
SCA15	-27.0	-30.8	-3.8	(-)
SCA17	n.a.	n.a.	n.a.	(-)
SCA18	n.a.	n.a.	n.a.	(-)
SCA19	-27.5	-32.1	-4.6	(-)
SCA20	-27.2	-31.9	-4.7	(-)
SCA23	n.a.	n.a.	na	(-)
SCA25	-27.3	-32.2	-4.8	(-)
**Viking/Norse**				
**Outer Hebrides**				
**Cille Pheadair,** South Uist				
KP1	-24.3	-27.2	-3.0	LC APAAs
KP2	-27.0	-29.6	-2.5	(-)
KP3	-26.9	-29.7	-2.8	(-)
KP4	-26.5	-31.3	-4.8	(-)
KP5M	-27.7	-30.4	-2.7	LC APAAs, LC DHYAs
KP5U	-27.3	-29.3	-1.9	LC APAAs
KP6	-27.2	-31.8	-4.7	LC APAAs
KP7	-27.0	-31.5	-4.5	(-)
KP8	-25.8	-30.4	-4.6	LC APAAs, LC DHYAs

KP9	-27.7	-30.9	-3.2	(-)
KP10	-26.2	-31.7	-5.5	LC APAAs
KP11	-24.8	-29.6	-4.8	(-)
KP12	-26.6	-28.9	-2.3	(-)
KP14	-27.6	-31.0	-3.4	(-)
KP19	-27.3	-31.2	-3.9	(-)
KP20	n.a.	n.a.	n.a.	LC APAAs, LC DHYAs
KP7vi	n.a.	n.a.	n.a.	(-)
KP 2v	n.a.	n.a.	n.a.	LC APAAs
**Bornais,** South Uist				
BN8	-27.6	-33.4	-5.8	(-)
BN8V	n.a.	n.a.	n.a.	(-)
BN28V	n.a.	n.a.	n.a.	LC APAAs
BN29	-27.0	-31.3	-4.3	(-)
BN33	-27.6	-33.6	-6.0	(-)
BN34	-26.3	-30.5	-4.2	(-)
BN34V	-25.0	-28.7	-3.6	LC APAAs
BN35	-25.4	-31.4	-6.0	LC APAAs, LC DHYAs
BN-35V	n.a.	n.a.	n.a.	LC APAAs
BN36	-27.3	-31.8	-4.5	LC APAAs, LC DHYAs
BN38	-25.7	-29.4	-3.7	LC APAAs
BN39	-27.6	-31.8	-4.2	LC APAAs
BN39V	n.a.	n.a.	n.a.	(-)
BN44	-26.9	-30.1	-3.2	(-)
BN45	n.a.	n.a.	n.a.	(-)
BN46	-26.1	-31.2	-5.1	(-)
BN46V	n.a.	n.a.	n.a.	(-)
BN47	-28.4	-33.6	-5.2	(-)
BN48	n.a.	n.a.	n.a.	(-)
BN49	-27.2	-31.6	-4.5	(-)
BN51	-26.5	-28.9	-2.5	(-)
BN52	-26.5	-29.1	-2.6	LC APAAs
BN52V	n.a.	n.a.	n.a.	(-)
BN53	-26.4	-29.4	-3.	LC APAAs
BN54	-26.5	-28.4	-2.0	(-)
BN55	-24.7	-28.2	-3.5	LC APAAs, LC DHYAs
BN90	-26.4	-29.9	-3.6	(-)
BN91	-24.3	-27.9	-3.6	LC APAAs, LC DHYAs
BN92	-27.6	-30.9	-3.4	(-)
BN93	-26.4	-30.9	-4.6	LC APAAs
BN94	-26.5	-28.7	-2.3	(-)
BN95	-26.6	-29.3	-2.7	(-)
BN96V	-26.9	-31.2	-4.3	(-)
BN97	-27.4	-30.3	-2.9	(-)

BN99	-29.0	-30.5	-1.5	(-)
BN100	-28.9	-30.5	-1.6	(-)
BN101	-28.1	-32.2	-4.2	(-)
BN103	-28.1	-30.1	-2.1	(-)
BN104	-27.3	-30.9	-3.7	(-)
BN105	-26.3	-31.7	-5.4	LC APAAs, LC DHYAs
BN107	-25.9	-32.3	-6.4	(-)
BN108	-27.5	-32.7	-5.2	(-)
BN110	-25.4	-25.9	-0.5	LC APAAs
BN111V	-26.8	-32.4	-5.6	(-)
BN115	-27.2	-31.0	-3.8	(-)
BN115V	-27.8	-30.6	-2.8	(-)
BN117	-27.3	-30.1	-2.8	(-)
BN118	-26.7	-30.1	-3.4	(-)
BN121	-26.8	-32.0	-5.2	(-)
BN122	-28.0	-30.8	-2.8	(-)
BN123	-26.5	-31.8	-5.4	(-)
BN123V	-26.9	-31.4	-4.5	(-)
BN124	-27.2	-29.8	-2.6	(-)
BN124V	-27.3	-29.7	-2.4	(-)
BN125	-28.2	-30.7	-2.5	(-)
BN126	-28.2	-32.5	-4.4	(-)
BN126V	-27.1	-32.4	-5.3	n.a.
BN130	-28.3	-33.1	-4.8	(-)
BN131	-26.8	-30.8	-4.0	(-)
**Shetland**				
**Jarlshof,** Mainland				
JAR1	-22.2	-21.8	0.4	LC APAAs
JAR2	-22.3	-22.5	-0.2	LC APAAs
JAR3	-22.5	-25.6	-3.1	LC APAAs
JAR5	-27.5	-30.5	-3.0	(-)
JAR6	-23.2	-25.3	-2.1	LC APAAs, LC DHYAs
JAR7	-23.6	-24.7	-1.1	LC APAAs, LC DHYAs
JAR8	-22.7	-22.4	0.3	LC APAAs, LC DHYAs
JAR12	-26.3	-28.1	-1.9	(-)
JAR13	-22.6	-24.3	-1.7	LC APAAs, LC DHYAs
JAR14	-26.1	-32.8	-6.6	LC APAAs, LC DHYAs
JAR14V	-24.7	-31.7	-7.0	LC APAAs
JAR15V	-22.2	-21.2	1.0	LC APAAs
JAR16	-27.4	-30.1	-2.7	LC APAAs
JAR17	-25.2	-29.0	-3.9	(-)
JAR18	-21.6	-23.6	-2.0	LC APAAs

JAR19	-23.8	-24.0	-0.2	LC APAAs, LC DHYAs
JAR21	-21.7	-21.4	0.3	(-)
JAR22	-24.1	-27.0	-2.9	LC APAAs, LC DHYAs
JAR22V	-25.6	-29.2	-3.6	LC APAAs
